# Fast Detection of Copper Content in Rice by Laser-Induced Breakdown Spectroscopy with Uni- and Multivariate Analysis

**DOI:** 10.3390/s18030705

**Published:** 2018-02-27

**Authors:** Fei Liu, Lanhan Ye, Jiyu Peng, Kunlin Song, Tingting Shen, Chu Zhang, Yong He

**Affiliations:** 1 College of Biosystems Engineering and Food Science, Zhejiang University, 866 Yuhangtang Road, Hangzhou 310058, China; lena_ye15@163.com (L.Y.); jypeng@zju.edu.cn (J.P.); kls19910820@163.com (K.S.); shentingtingstt@163.com (T.S.); chuzh@zju.edu.cn (C.Z.); yhe@zju.edu.cn (Y.H.); 2 Key Laboratory of Spectroscopy Sensing, Ministry of Agriculture, Hangzhou 310058, China

**Keywords:** laser-induced breakdown spectroscopy (LIBS), rice, copper content, univariate analysis, multivariate analysis

## Abstract

Fast detection of heavy metals is very important for ensuring the quality and safety of crops. Laser-induced breakdown spectroscopy (LIBS), coupled with uni- and multivariate analysis, was applied for quantitative analysis of copper in three kinds of rice (Jiangsu rice, regular rice, and Simiao rice). For univariate analysis, three pre-processing methods were applied to reduce fluctuations, including background normalization, the internal standard method, and the standard normal variate (SNV). Linear regression models showed a strong correlation between spectral intensity and Cu content, with an $R^{2}$ more than 0.97. The limit of detection (LOD) was around 5 ppm, lower than the tolerance limit of copper in foods. For multivariate analysis, partial least squares regression (PLSR) showed its advantage in extracting effective information for prediction, and its sensitivity reached 1.95 ppm, while support vector machine regression (SVMR) performed better in both calibration and prediction sets, where $R_{c}^{2}$ and $R_{p}^{2}$ reached 0.9979 and 0.9879, respectively. This study showed that LIBS could be considered as a constructive tool for the quantification of copper contamination in rice.

## 1. Introduction

Food safety has become a critical issue in public opinion, scholarly research, professional management, and government regulations worldwide, especially for China where the whole society has arguably been threatened by it more than by any other problems [1]. Rice, as a major source of calories and mineral nutrients for Chinese people, is of concern to the public because of heavy metals contamination. Copper is ranked as the fourth most contaminating heavy metal in farmland, for it can contribute to the generation of highly-reaction oxygen species which might cause damage to cells with excessive concentration [2]. Previous study has shown that copper accumulation in plants mainly resulted from root uptake [3], which implied that excessive copper content might be caused by polluted soils and irrigation waters. It was found that the average concentration of Cu in paddy soil was 10 times higher than the Grade II environmental quality standard for soils in some parts of China (GB15618-1995) [4]. The long-term use of wastewater irrigation also led to significantly higher concentrations in soils than reference ones [5]. According to the tolerance limit of copper in food (GB 15199-1994), the concentration of Cu in grain is not allowed to be higher than 10 mg/kg. Although no evidence showed that the concentration of Cu exceeded the national maximum allowable levels in rice, it was suggested that it might exceed the reference oral dose for adults and children, based on the estimated daily intake by human beings [6].

The main spectral analytical methods for the detection of copper content in food are flame atomic absorption spectrometry (FAAS) [7], inductively-coupled plasma optical emission spectrometry (ICP-OES), and inductively-coupled plasma mass spectrometry (ICP-MS) [8]. However, these traditional methods, when analyzing copper content in solid samples, required complex pretreatment. The results of detection are sensitive to the processes of pretreatment. Laser-induced breakdown spectroscopy (LIBS) is a recently developed analytical technique for quantitative element analysis in food. A sample is heated and stimulated by a powered laser, leading to the formation of a plasma above the surface [9]. The wavelength of emission light from the plasma and the corresponding intensity reflects the elementary composition and content in samples [10]. Minimal or no sample preparation is one of its main advantages, which could free us from complex processes of digestion and save time. Along with its advantages of fast analysis speed, multiple elemental measurements, and the capability of remote sensing, LIBS may meet the demand of real-time measurement in agriculture [11]. Some preliminary studies concerning the quantitative analysis of Cu in the field of food detection has been published, including food supplements [12], nutrient elements [13], and analysis of contaminants [14].

However, the accuracy and precision of quantitative analysis can be affected by atmospheric conditions, and the physical and chemical properties of samples [15]. Additionally, the pulse-to-pulse fluctuations in LIBS can be very large with the relative standard deviation (RSD) of laser pulse energy up to 60% [16], and the analysis uncertainties are generally 4% to 15% with proper data-processing methods [17]. On the other hand, since the physical and chemical properties of the target can influence the processes of sample ablation, plasma formation, and other associated processes [18], it can affect the composition of the laser-induced plasma and result in matrix effects [19]. With respect to the physical properties, sample preparation, like grinding, screening, and tablet pressing, were routinely necessary for a flat surface and a uniform microstructure [20]. With respect to the chemical properties of samples, matrix effects include both spectral and non-spectral interferences. In our experiment, the chosen Cu characteristic spectral lines did not overlap with other emission lines, so that non-spectral interferences of matrix effects were the main problem and needed to be eliminated. Otherwise, the calibration curves for different samples would be significantly different and subsequently influence the results of quantitative analysis [21].

Univariate analysis is a traditional quantitative analysis method in LIBS analysis. However, this method is prone to be affected by the matrix. According to the previous studies, analytical signals could be corrected through pre-processing methods [22], like internal standards and multivariate signal correction. Twelve different types of normalizations have been used and proven as useful methods to reduce the differences among sample matrices [23]. Among them, internal standard normalization is a conventional procedure, where the internal standards can be the constituent element of the target samples, as well as a buffer gas [24]; but it may be quite difficult to find a suitable element as an internal standard [25]. Background normalization has proved to be an effective method for the problem of the matrix effect, since the intensity of the continuum radiation directly relates to the plasma [16]; however, it may restrict the laser energy in a small range [26]. Standard normal variate (SNV) can be used to minimize unwanted variations caused by particle size distribution [27], as well as to deal with multivariate analysis [28].

Multivariate analysis has its advantage in extracting chemical information from spectra, while the performance depends on the algorithm used for calibration. Partial least squares regression (PLSR), which is one of the most commonly used multivariate analysis for chemical analysis, has been widely applied to LIBS analysis, like jewelry [29], alloys [30], and plants [31]. The factors related the variation in the response measurements, are regressed against the properties of interest and applied for predicting the interested properties of testing samples [32]. This method has been proven as a good model to minimize the differences of different types of sugar cane varieties [33]. Support vector machine regression (SVMR) is a quite new method for LIBS analysis. This is a non-linear regression, which has been widely used in the analysis of VIS/NIR recently [34]. This potential method has been applied for agricultural soils with mobile LIBS, showing its abilities in utilizing the most spectral information to search the relationships within different variables [35].

The aim of our study is to demonstrate LIBS as a practical method for the fast detection of copper content in rice. In this experiment, uni- and multivariate analyses were employed to establish the calibration model for quantitative analysis. For univariate analysis, three pre-processing methods were used to eliminate the fluctuations of experimental condition. For multivariate analysis, two different algorithms were used and compared to diminish the non-spectral interferences of matrix effects.

## 2. Materials and Methods

### 2.1. Rice Material and Sampling

Three kinds of rice commonly found in local supermarkets were used in this experiment, which were Simiao rice (originating from Guangdong, China), Jiangsu rice (originating from Jiangsu, China) and regular rice (originating from Shanghai, China). Samples with different copper contents were prepared in the laboratory with the treatment of a copper (II) sulfate pentahydrate solution [36].

Copper (II) sulfate pentahydrate (${\mathrm{CuSO}}_{4}\cdot 5H_{2}O$, Hushi, Shanghai, China, AR) was applied to prepare a series of solutions, where Cu concentrations were 25 mg/L, 50 mg/L, 100 mg/L, 200 mg/L, 400 mg/L, 600 mg/L, 800 mg/L, and 1000 mg/L. Rice was evenly divided into nine groups for each kind, one as the control group and the rest as treatment groups to obtain rice samples with a series of Cu concentrations. Each group was soaked in the solution with certain Cu concentration at 4 °C for 48 h, and then rinsed three times with ultrapure water. All the samples were dried at room temperature, respectively milled by high-speed pulverizer (FW100, TAISITE, Tianjin, China), and then filtered through 200-mesh screens. The milling and filtering processes made the ground sample of each group a uniform mixture to ensure the consistency of Cu content within each group. These ground samples were made into pellets by a tablet compressing machine (FY-24, SCJS, Tianjin, China), 1.5 cm in diameter, under a pressure of 25 kN for 1 min. A total of 270 samples were prepared with 10 parallel samples for each group.

### 2.2. LIBS Experimental

**Figure 1:**
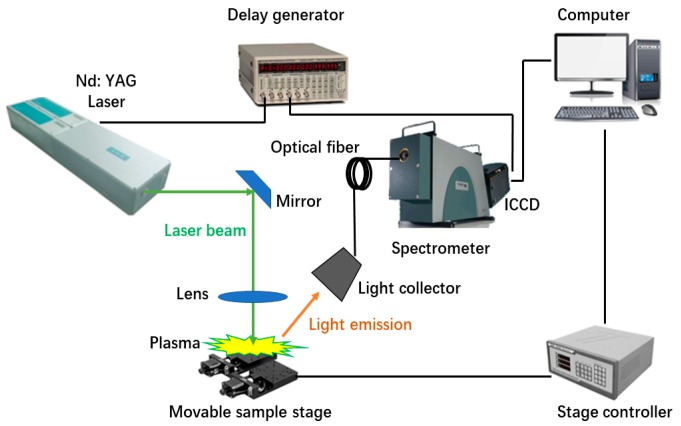
The structural diagram of the LIBS system.

The experimental setup mainly consists of a pulsed laser (Vlite 200, Beamtech, Beijing, China), a delay generator (DG645, Stanford Research Systems, Sunnyvale, CA, USA), optics, a movable sample stage (TSA50-C, Zolix, Beijing, China) with controller (SC300, Zolix, Beijing, China), a spectrograph (ME5000, Andor, Belfast, UK), an ICCD camera (DH334-18F-03, Andor, Belfast, UK), and a computer. Figure 1 shows the structure of the LIBS system used in the experiment. A high-power laser pulse is focused on the surface of the sample through a set of optics. The interaction of a laser beam with matter is complex and not fully understood yet, but it will lead to the formation of a plasma with a high temperature above the sample surface. This evaporated plasma contains many excited species whose light emissions are later collected and analyzed [9].

In our experiment, the Nd:YAG laser was used as an excitation source, delivering pulse energy of 60 mJ with a pulse width of 8 ns at a wavelength of 532 nm and a repetition rate of 1 Hz. The laser beam was focused on the samples with a 100 mm focal length optical lens, producing an ablation spot of 500 μm in diameter. In addition, acquisition delay time was 3 μs and gate width 10 μs, optimized by pre-experiment to get an ideal signal-to-background ratio (SBR). This experiment was performed in an atmospheric environment. A movable sample stage was controlled by a stepper motor to accurately control the position and movement of samples. The accumulation of five laser pulses was taken for each spectrum, and sixteen LIBS spectra were acquired in sixteen different positions for each pellet. These sixteen spectra were further averaged to eliminate shot-to-shot fluctuations. As a result, each spectrum revealed the spectral information of its corresponding sample.

### 2.3. Determination of the Reference Value of Copper Content

The concentration of Cu in all rice samples, determined by flame atomic absorption spectrometry (FAAS) analysis (iCE 3500, Thermo Scientific, Waltham, MA, USA), were used as reference values [37]. The detection procedures were based on Chinese National Standard GB/T 5009.13-2003. Standard samples of rice (GBW10010) and French bean (GBW10021) were used in order to assure the accuracy of the results. All glassware was cleaned by soaking in 20% ${\mathrm{HNO}}_{3}$, and rinsed with ultrapure water and dried. For dry digestion of samples, portions (0.5 g) of each rice sample were weighed into crucibles, carbonized for 20 min and then dry-ashed at 550 °C for 3 h. After cooling to room temperature, residuals were dissolved by 5 mL diluted HCl. If necessary, an additional 100 μL of conc. nitric acid and heating gently can help to dissolve the residuals. The solution was transferred to a 25-mL volumetric flask and diluted with ultrapure water, and then filtered. A blank sample was prepared in the same way as a comparison.

### 2.4. Statistical Analysis

Data were processed via The Unscrambler X (ver. 10.4, Camo Software, Oslo, Norway) and MATLAB (ver. 2012, MathWorks, The Natick Mall, MA, USA). 

For univariate analysis, peak height and peak area were first obtained with Voigt profile fitting, which has been used for separating from lines of interest from nearby emission lines [38]. Three pre-processing methods, including internal standard methods, background normalization, and standard normal variate (SNV), were applied separately and compared. The coefficient of determination ($R^{2}$) for linear regression was the indicator to evaluate the performance of the pre-processing method.

For multivariate analysis, all the LIBS spectra were divided into two sets: by sorting the samples from lowest to highest concentrations according to the reference values, two in every three samples were assigned to a training set (for calibration), and the rest to a validation set (for prediction). Principal component analysis (PCA) is a classic technique used for feature extraction and data representation [39]. This method provides visual results of relationships between samples and variables, and insights into sample homogeneities and heterogeneities at the same time [40]. It is widely used as a method of classification [41] and dimensionality reduction [42] in LIBS. In this experiment, PCA was used to visualize the differences among different kinds of rice, and served as a dimensionality reduction method. The partial least squares (PLS) algorithm is a factor analysis method, which is desirable to acquire more predictive information in the first few factors [43]. In this case, partial least square regression (PLSR) was used here to construct a multivariate linear model, as well as for quantitative prediction. Support vector machine regression (SVMR) is derived from support vector machine (SVM) [44]. It does well in non-linear regression for high-dimensional datasets [45], and was applied to construct a non-linear regression model here. There are two main parameters in SVMR, which includes the penalty parameter C and the kernel function parameters, such as γ for the radial basis function (RBF) kernel [46]. 

The root mean squared error for calibration (RMSEC), cross-validation (RMSECV), and prediction (RMSEP), and the coefficient of determination for cross-validation ($R_{\mathrm{cv}}^{2}$) and prediction ($R_{p}^{2}$), were used as indicators to evaluate the performance of models. RMSECV was used to optimize the model’s parameters [1], and preferable parameters were used when the value of RMSECV reached its minimum. In order to avoid over-fitting, a 10-fold cross-validation was performed to optimize the parameters [47] until the values of RMSEC, RMSECV, and RMSEP were the closest.

## 3. Results and Discussion

### 3.1. Spectral Characterization of Rice Samples

**Figure 2:**
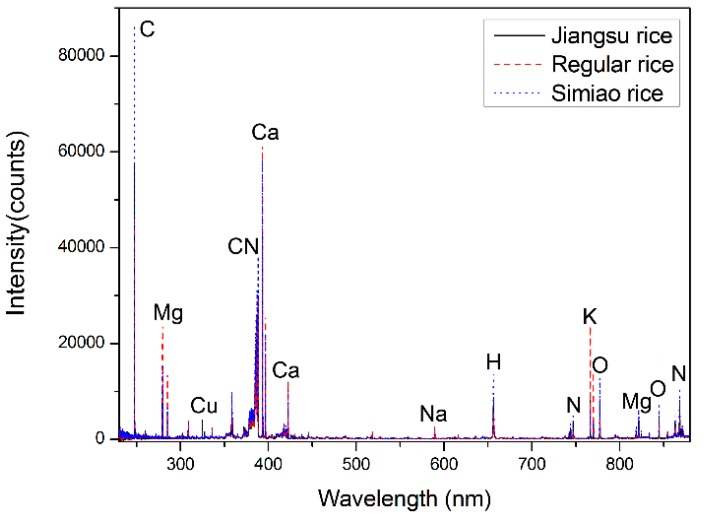
LIBS spectra of three different kinds of rice. Raw spectra of three different kinds of rice without extra added Cu.

LIBS is a form of element analytical spectroscopy, reflecting the emissions of excited atoms and ions from a produced plasma [19]. The average spectra of three kinds of rice samples are illustrated in Figure 2, showing the similarity of elemental composition in rice. According to the NIST database, the main spectral emission lines corresponded to C (247.856 nm), Mg (279.553 nm, 285.213 nm, and 821.304 nm), Cu (324.754 nm and 327.396 nm), CN (388.246 nm), Ca (393.366 nm and 422.673 nm), Na (589.592 nm), H (656.277 nm), N (742.364 nm, 744.229 nm, 746.831 nm and 868.340 nm), K (766.490 nm and 769.896 nm), and O (777.194 nm and 844.636 nm). In this case, a chemometric data analysis method was needed for further detailed analysis. Samples, without adding extra Cu, were used to distinguish the differences among three kinds of rice, i.e., 10 samples for each kind and 30 samples in total.

**Figure 3:**
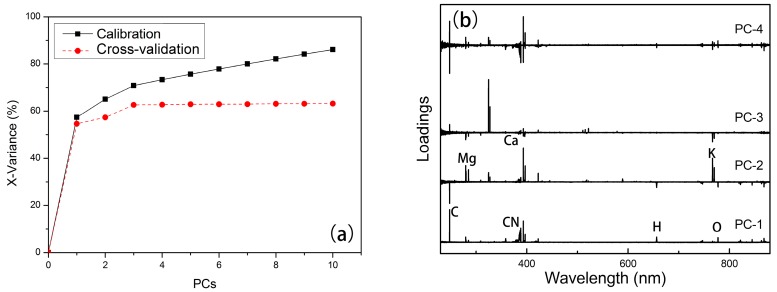
Results of PCA. (**a**) Explanation of X-variance; and (**b**) PCA loadings of PC1-PC4.

The LIBS full spectra were analyzed by PCA. The total number of rows (30) and columns (22,036) corresponded to the number of samples and the spectral wavelength, respectively. The maximum number of PCs was set as 10 when calculating. The loadings (see Figure 3b) showed the main distinguishing elements of the spectra, e.g., C at 247.856 nm, Mg at 279.553 nm and 285.213 nm, K at 766.490 nm, and Ca at 393.366 nm and 422.673 nm.

**Figure 4:**
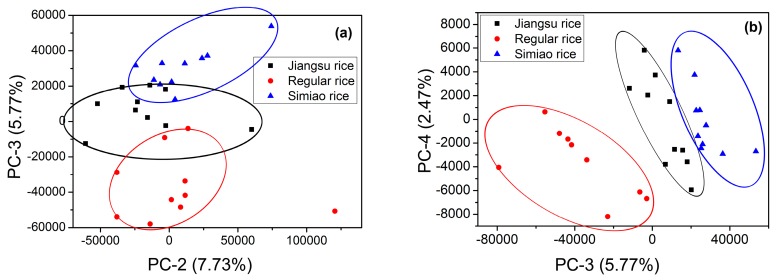
Scatter plots of PCA scores. (**a**) Scores plot of PC2 vs. PC3; and (**b**) scores plot of PC3 vs. PC4.

Although PC-1 had the largest possible variance, the differences of these three kinds of rice were not significant using PC-1. According to loadings plot, PC-1 was mainly concerned with the information of C, H, and O, and the content of these elements might not have large differences among different kinds of rice. On the other hand, PC-2, PC-3, and PC-4 also included the information of Mg, K, etc., which might be the reason leading to the diversity. According to the scores plot of PC-2 versus PC-3 (see Figure 4a), differences among three kinds of rice is feasible, while clusters are overlapped, especially for Jiangsu rice and Simiao rice. Samples are well separated in Figure 4b.

The dispersion of samples for certain kinds of rice could also be seen in the plots. In the LIBS spectra, PCs revealed the elemental components of samples. The dispersion might show the individual differences of samples. For another, inhomogeneous microstructure of each sample within each pellet may contribute to this phenomenon [48].

### 3.2. Comparison the Results of Univariate Analysis with Different Pre-processing Methods—Univariate Calibration

In addition to the three different kinds of rice, data involving all three kinds of rice was also considered as a set for quantitative analysis. All of the raw data were processed by baseline correction beforehand, to reduce the influence of the background. Spectral lines at 324.754 nm and 327.396 nm of Cu were used for the following univariate analysis. Reference values of Cu concentrations were detected by FAAS, and results and uncertainties are shown in Table 1.

**Figure 5:**
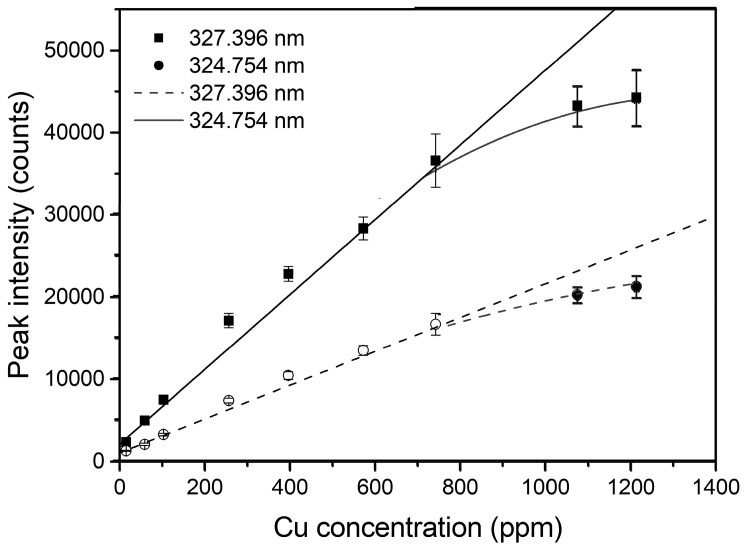
Scatterplots and curve-fitting results of reference Cu concentrations of Simiao rice versus the peak intensity of Cu I 324.754 nm and 327.396 nm. The reference concentration of Cu was obtained by FAAS.

Figure 5 shows the relationship between Cu concentrations of Simiao rice and their corresponding peak intensities at 324.754 nm and 327.369 nm. According to the curve-fitting results, self-absorption phenomenon was evident when the reference Cu concentration was larger than 1000 ppm. However, no feasible self-absorption could be found at the corresponding spectral lines. According to the previous study, a pronounced non-linearity in the calibration function at increasing concentration (greater than approximately 1000 ppm), with or without a feasible occurrence of self-absorption on the spectra, was caused by the complex interaction mechanism between the atoms/ions and the radiation which is emitted and successively reabsorbed [49]. In this experiment, we would simply focus on the data where Cu concentrations are smaller than 1000 ppm.

In addition to natural broadening, Doppler broadening, instrumental broadening and collision broadening are the main line broadening mechanisms in LIBS [50]. The Doppler broadening and instrumental broadening lead to a Gaussian line profile while the latter leads to a Lorentz profile. Hence, the actual measured line profile might be the convolution of these two kinds of profiles. In that case, Voigt profile was applied, and it was fitted well with the raw data of spectra ($R^{2}$ more than 0.99). Then, the peak area was calculated through integrating the peak intensity versus wavelength, and the continuous background was subtracted through linear fitting. The results of linear regressions using peak height and peak area are shown in Table 2. For Jiangsu Rice and Simiao Rice, the performance of this linear regression model was pretty good with $R^{2}$ more than 0.98, while regular rice performed worse, with an $R^{2}$ just over 0.90. In general, Voigt profile fitting performed no improvement or offered even worse results, compared with the values of $R^{2}$ with peak intensities of the corresponding spectral lines. This indicated that Cu I at 324.754 nm and 327.396 nm were not influenced by nearby emission lines. The peak intensity of the spectrum is sufficient to express the linear relationship with Cu concentration in rice, which would be used for the following data pre-processing.

The calculated coefficients of variation of peak intensities for each kind of rice varied from 4– 25% (n = 10), which might affect the performance of linear regression. According to the previous study [51], the intensity of the signal correlated with the amount of the ablated matter to some degree. The fluctuation of pulse energy is one of the reasons that cause this problem [52], and the RSD for shot-to-shot variation could reach up to 60% in LIBS [16]. During the experiment, we also found that the ablated craters became larger with higher metal element concentrations, which would absolutely make an impact on the signal intensities and subsequent linear regressions. Additionally, the diversities of different kinds of rice (which we discussed in Section 3.1) might contribute to the fluctuations of intensity, as well. The most common methods to reduce fluctuations for quantitative analysis were the “internal standard” method and the “background normalization” [53]. 

The traditional “internal standard” method chooses an element or an external element, of which the concentration is known beforehand. In LIBS, instead, the internal standard element could be the main element with an almost constant content [25]. Carbon was an ideal choice as the internal standard element in this experiment, and C II at 247.856 nm was easily recognized in the spectra. The results of linear regression showed that analytical lines at Cu I 324.754 nm normalized by C II at 247.89 nm provided better linearity of the calibration curve for each kind of rice, as well as for the whole samples, especially for regular rice with $R^{2}$ reaching 0.9717, while the results were worse for Cu I at 327.396 nm (except for Jiangsu rice).

“Background normalization” is another effective way for correcting this problem, which is based on the spectral intensity normalized by the continuous background of the laser plasma. This method is preferred when no ideal internal standard can be found [53], and has been proved to be reliable and accurate in reducing fluctuations to the external error sources [54].The value of the background was an averaged intensity of a spectral range near the interested spectral line, within which no other strong emission lines could be found. Overall, this pre-processing method slightly improved the linear regression results for Cu I at 324.754 nm and slightly worsened the results for Cu at 327.396 nm, except Regular rice of which $R^{2}$ improved to 0.9717 and 0.9769, respectively. 

The standard normal variate (SNV) method could take the place of normalization with a spectral line when it is difficult to find a constant element, as well as be used to eliminate the fluctuations of experimental conditions (external influences), including temperature, pressure, and pulse energy. According to Table 2, this was an effective method to alleviate the external interferences, as the $R^{2}$ of linear regression improved for almost every single kind of rice (except Cu I at 327.396 nm for Simiao rice). However, it made little contribution to the matrix effect (internal interferences), as no significant improvement of the values of $R^{2}$ were observed. This was in accordance with the previous study, saying that SNV was only suitable for analysis based on the same matrix, for the SNV transformation depends on the mean and standard deviation of the entire spectrum, which can easily be influenced by the material [28].

For all three kinds of rice, however, these pre-processing methods had little impact on the performance of linear regression. It was implied that the fluctuations of the experimental conditions were not the main factor which could influence the quantitative analysis of copper content in different kinds of rice.

**Figure 6:**
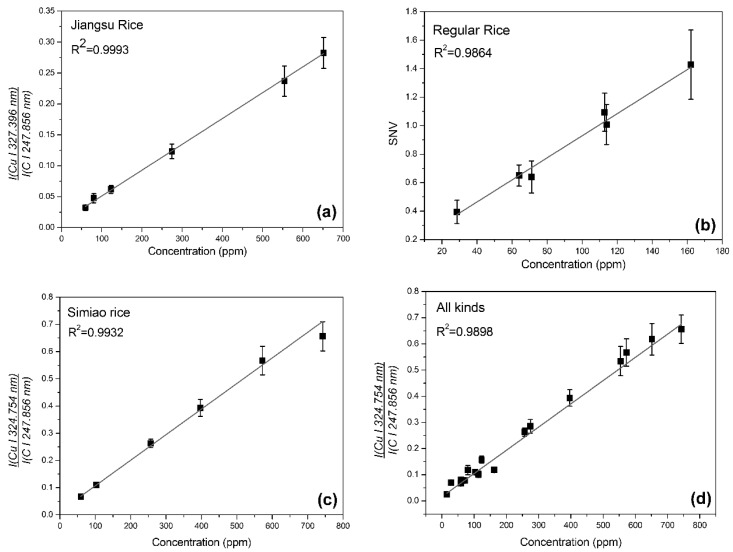
The best univariate calibration curves. (**a**) Jiangsu rice: I (Cu I 327.396 nm)/I (C I 247.856 nm); (**b**) regular rice: Cu at 327.396 with SNV processed; (**c**) Simiao rice: I (Cu I 324.754 nm)/I (C I 247.856 nm); and (**d**) the total samples: I (Cu I 324.754 nm)/I (C I 247.856 nm).

The best results of calibration curves were shown in Figure 6. The preferable results were processed with internal standard C at 247.856 nm, except for regular rice. The limit of detection (LOD) was calculated based on the 3$\sigma_{B}$-IUPAC definition [18]:

(1) $$\mathrm{LOD}=\frac{3\sigma_{B}}{S}$$

where$\;\sigma_{B}$ is the standard deviation of the background, and S is the slope of the calibration line. The best LOD were obtained at Cu 324.754 nm with the process of SNV, which were 4.63 ppm for Jiangsu rice, 8.13 ppm for regular rice, 4.87 ppm for Simiao rice and 4.64 ppm for all kinds.

### 3.3. Comparison the Results of Multivariate Analysis with Different Algorithms (PLS and SVMR)

**Figure 7:**
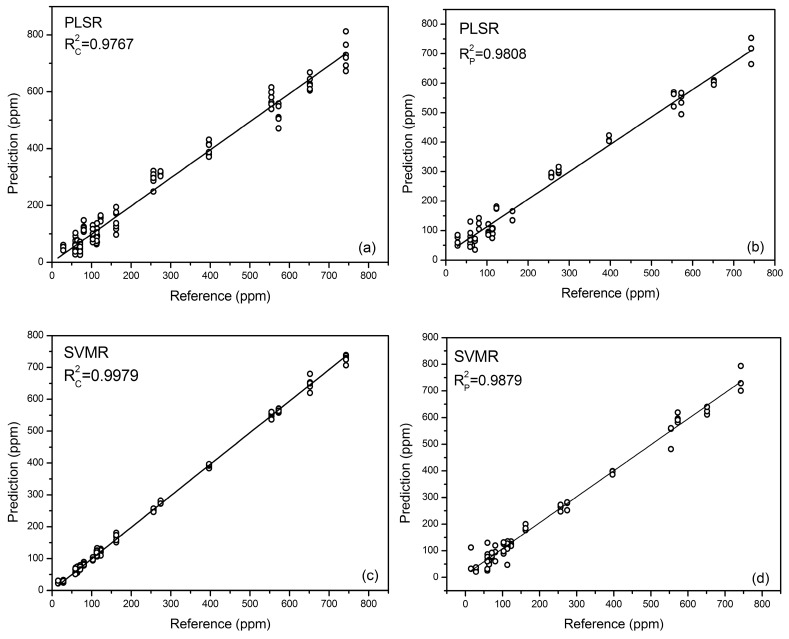
Calibration and prediction for multivariate analysis. (**a**) The calibration result of PLSR; (**b**) the prediction result of PLSR; (**c**) the calibration result of SVMR; and (**d**) the prediction result of SVMR.

Aiming at alleviating the problem of matrix effects, multivariate analysis was applied to all three kinds of rice samples. In this experiment, the PLSR linear method and the SVMR nonlinear method were employed to establish the calibration models. The results for multivariable analysis of three kinds of rice samples are shown in Table 3 and Figure 7.

For the PLS model, the number of latent variables was optimized to three when the minimal RMSECV was achieved, explaining more than 97% of the X-variance. The performance of PLSR was comparable with univariable analysis, with $R_{p}^{2}$ of 0.9808 and RMSEP of 33.2252 mg/kg. The sensitivity of PLS is 1.95 ppm, which was calculated using the net analyte signal (NAS) [55].

**Figure 8:**
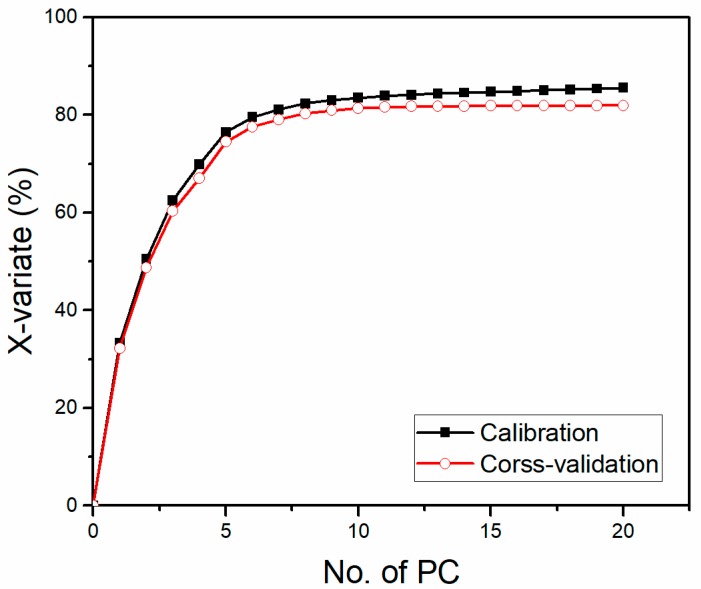
Explanation of the X-variate for PCA.

For the SVMR model, the performance was not so ideal (see Table 3) with an $R_{\mathrm{cv}}^{2}$ of 0.8816 and $R_{p}^{2}$ of 0.8858. The distinct differences of RMSEC, RMSECV, and RMSEP implied the over-fitting problem of the model. This might be due to too many independent variables of the spectral data [1]. In that case, PCA was applied here as a data reduction method to obtain a more effective regression model. According to Figure 8, the first 17 PCs were used which explained more than 85% variance of the data. Relatively good performance of SVMR could be acquired, where $R_{c}^{2}$ and $R_{p}^{2}$ were 0.9979 and 0.9879, respectively, and the problem of over-fitting was relieved a great deal, for the close value of RMSECV and RMSEP [47]. The calibration result showed SVMR as an effective method dealing with matrix effects. However, the problem of overfitting could not be avoided, comparing the results of calibration and cross-validation for SVMR (see Figure 7).

Both algorisms could eliminate matrix effects to some degree. PLS might be more reliable compared with SVMR, because it had similar performances of calibration and validation. SVMR, on the other hand, was easy to be overfitted, which led to the relatively larger RMSECV and RMSEP, especially before the process of data reduction. Hence, some feature extraction methods, such as PCA, were recommended before SVMR was applied. 

## 4. Conclusions

Relatively easy sample preparation is one of distinctive advantages of LIBS over other established spectrochemical methods, like FAAS. For one thing, the processes are easy and repeatable, including drying, grinding, and tablet pressing. The instruments needed for sample preparation are quite common, and no aggressive chemicals, like nitric acid or hydrogen peroxide, are needed. For another, in our experiment, 270 samples were made in total. We required about nine hours of preparation for LIBS, during which only around 100 samples could be prepared using microwave digestion. Additionally, samples with certain requirements could be made with easy methods in the laboratory, like soaking samples in salt solutions with different concentrations of heavy metal elements [56], which would be very meaningful for further studies.

In this experiment, results of PCA analysis showed the different contents of nutrient elements (e.g., C, K, and Mg) in different kinds of rice. This implied the feasibility of LIBS as a method for the classification for different kinds of rice, as well as evaluating product quality. The distinguished differences among different kinds of rice samples also indicated the existence of matrix effects, and the need for proper data processing in order to acquire a reliable and accurate analytical model for the determination of Cu concentration in rice.

For univariate analysis, a significant non-linearity suggested that Cu concentrations were no more than 1000 ppm. Strong linear relationships could be seen between Cu concentrations of samples and the corresponding characteristic peak intensities of the spectra. Cu at 324.754 nm is more preferable for quantitative analysis, due to its relatively higher intensity and lower LOD. In general, pre-processing methods used in this experiment, i.e., internal standard method, background normalization, and SNV, could be used to correct the variation of experimental conditions, e.g., the fluctuation of pulse energy. The internal standard method might be more preferable than the other two, and C at 247.856 nm could be an ideal choice as an internal standard. However, these three pre-processing methods have little effect to the differences of the target, itself, such as matrix effects.

For multivariate analysis, PLS showed its high efficiency in extracting effective information from the vast spectral data, and obtaining good results for modeling and prediction. Additionally, the sensitivity of PLS reached as low as 1.95 ppm, while PCA-SVMR showed its capability for reducing the influence of the matrix effect, as well as offering preferable results with $R_{c}^{2}$ reaching 0.9979 and $R_{p}^{2}$ reaching 0.9879.

Good results for both qualitative and quantitative analysis showed that LIBS could be an efficient and convenient method for classification, as well as quantification of copper content in rice. RSDs for univariate analysis with data pre-processing were lower than 15% with data pre-processing and were lower than 12% for multivariate analysis, which were more than 20% for raw data. The presented results provided the first proof-of-principal data for fast detection of heavy metals in food.

## Figures and Tables

**Table 1:** Reference values of Cu concentrations and uncertainties detected by FAAS

Regular Rice	Content (ppm)	Jiangsu Rice	Content (ppm)	Simiao Rice	Content (ppm)
1	5.68 ± 0.06	1	7.60 ± 0.11	1	5.10 ± 0.06
2	28.54 ± 0.78	2	59.48 ± 0.58	2	59.07 ± 1.18
3	46.35 ± 0.57	3	80.45 ± 1.53	3	103.18 ± 1.07
4	63.99 ± 1.17	4	123.09 ± 1.56	4	256.52 ± 4.43
5	71.09 ± 1.02	5	274.34 ± 4.22	5	396.65 ± 4.91
6	113.94 ± 2.26	6	554.35 ± 7.78	6	572.53 ± 9.72
7	116.84 ± 4.94	7	651.68 ± 8.07	7	742.62 ± 11.13
8	1162.10 ± 34.86	8	1104.76 ± 19.10	8	1074.92 ± 11.06
9	1668.75 ± 33.36	9	1115.28 ± 13.49	9	1213.76 ± 15.41

**Table 2:** The results for univariate calibration with different pre-processing methods.

Analytical Signal	$R^{2}$			
		Regular Rice	Simiao Rice	All Kinds
324.754 nm				
Peak intensity	0.9962	0.9051	0.9880	0.9875
Peak intensity/Background	0.9971	0.9601	0.9884	0.9845
Voigt profile (peak height)	0.9958	0.8985	0.9863	0.9853
Voigt profile (area)	0.9941	0.9033	0.9822	0.9807
Peak intensity/C I 247.856 nm	0.9989	0.9717	0.9932	0.9898
SNV	0.9984	0.9719	0.9895	0.9873
327.396 nm				
Peak intensity	0.9985	0.9303	0.9918	0.9884
Peak intensity/Background	0.9964	0.9769	0.9893	0.9829
Voigt profile (peak height)	0.9984	0.9227	0.9920	0.9886
Voigt profile (area)	0.9985	0.9183	0.9912	0.9830
Peak intensity/C I 247.856 nm	0.9993	0.9125	0.9880	0.9863
SNV	0.9986	0.9864	0.9889	0.9872

**Table 3:** The results for multivariate analysis for total samples with different modeling algorithms.

Algorithm	Calibration				Validation	
	$R_{c}^{2}$	RMSEC	RMSCV	$R_{cv}^{2}$	$R_{p}^{2}$	RMSEP
PLSR	0.9767	34.8186	38.1774	0.9727	0.9808	33.2252
SVMR	0.9929	33.5980	121.6671	0.8816	0.8858	84.0770
SVMR (data reduction)	0.9979	11.1120	26.6166	0.9866	0.9879	24.7755
